# A review of adipose-derived mesenchymal stem cells‘ impacts and challenges: metabolic regulation, tumor modulation, immunomodulation, regenerative medicine and genetic engineering therapies

**DOI:** 10.3389/fendo.2025.1606847

**Published:** 2025-05-29

**Authors:** Lihong Wang, Xiaorui Jiang, Fen Zhao, Ping Duan, Zhengguang Li, Yiqiao Luo

**Affiliations:** ^1^ Chengdu Integrated TCM and Western Medical Hospital, Department of Oncology, Chengdu, Sichuan, China; ^2^ Cancer Center, West China Hospital, Sichuan University, Chengdu, China

**Keywords:** adipose-derived mesenchymal stem cells (ADSCs), metabolic regulation, tumor microenvironment (TME), metabolic syndrome, immunomodulation, drug delivery systems

## Abstract

Adipose-derived mesenchymal stem cells (ADSCs), multipotent stromal cells abundant in adipose tissue, exhibit remarkable plasticity in modulating systemic metabolism, inflammatory responses, and immune homeostasis. Their bidirectional interactions with the tumor microenvironment (TME) position them as both accomplices and antagonists in cancer progression, offering unique therapeutic opportunities. ADSCs also hold significant potential for clinical application in the fields of regenerative medicine, tissue engineering, and gene engineering. This review synthesizes the impacts and challenges of ADSCs involving metabolic regulation, tumor modulation, immunomodulation, regenerative medicine and genetic engineering therapies—while elucidating underlying molecular mechanisms and signaling pathways, clinical studies, applications and challenges.

## 1 Introduction

Adipose-derived mesenchymal stem cells (ADSCs), a subset of mesenchymal stem cells (MSCs), are multipotent stromal cells isolated from the stromal vascular fraction (SVF) of adipose tissue (1). These cells share fundamental molecular and functional similarities with other mesenchymal stem cells (MSCs), such as bone marrow-derived mesenchymal stem cells (BMSCs), including expression of characteristic surface markers (CD105, CD44, CD73, CD90, CD146) and absence of hematopoietic lineage markers (CD13, CD19, CD45, HLA-DR) (2). Like other MSCs, ADSCs exhibit self-renewal capacity and multilineage differentiation potential (3). Beyond differentiation, ADSCs possess immunomodulatory properties mediated through paracrine secretion of anti-inflammatory cytokines (e.g., IL-10, TGF-β) and direct cell-cell interactions making them a promising candidate for regenerative medicine and reconstructive therapies (4, 5). Furthermore, adipose-derived mesenchymal stem cell exosomes (ADSC-Exos), enriched with diverse bioactive molecules including miRNAs, proteins, and lipids, significantly contribute to anti-inflammatory responses and immune regulation, while their cell-free transplantation circumvents risks of immune rejection and tumorigenesis associated with traditional stem cell therapies (6). ADSCs are easily accessible and minimally invasive procedures such as liposuction (7, 8). And their abundance in adipose tissue, coupled with low donor-site morbidity, positions ADSCs as a clinically viable resource for applications including anti-inflammatory, immunomodulatory, metabolic regulation and therapeutic potential (9, 10). Current research on ADSCs remains predominantly confined to elucidating basic molecular mechanisms, with insufficient preclinical validation and clinical translation to establish systematic frameworks or practical guidelines. To address these limitations, this study adopts a translational pipeline spanning “basic medical research (molecular pathways) → preclinical testing (*in vitro*/animal models) → clinical trials → therapeutic applications” to comprehensively evaluate ADSCs’ roles and challenges in metabolic regulation (diabetes, obesity), tumor modulation (pro-/anti-tumor effects), immunomodulation (autoimmune diseases, transplantation), regenerative medicine (tissue repair, osteoarthritis, cardiac/neural regeneration), and genetic engineering therapies.

## 2 Metabolic regulation

### 2.1 Diabetes mellitus

Diabetes mellitus (DM), encompassing both type I and type II forms with distinct pathogenic mechanisms, is characterized by persistent hyperglycemia; inadequate glycemic control frequently leads to severe complications such as retinopathy, neuropathy, and vasculopathy, all of which profoundly compromise patient health. Type 1 diabetes mellitus (T1DM) is primarily characterized by autoimmune-mediated destruction of pancreatic β-cells, while type 2 diabetes mellitus (T2DM) manifests as a triad of insulin resistance in target organs (skeletal muscle, liver, and adipose tissue), chronic low-grade inflammation driven by pro-inflammatory cytokines (e.g., TNF-α, IL-6) secreted from macrophages and other immune cells, and progressive β-cell dysfunction due to glucolipotoxicity and endoplasmic reticulum stress (11). ADSCs-mediated improvement of hyperglycemia may encompass islet β-cell regeneration via differentiation into insulin-producing cells or promotion of endogenous β-cell proliferation, modulation of hepatic metabolism toward enhanced glucose utilization, attenuation of chronic inflammation through anti-inflammatory cytokine secretion, and amelioration of insulin resistance (IR) in peripheral tissues via regulation of lipid homeostasis and insulin signaling pathways (12–15). ADSCs transplantation facilitates the restoration of islet function through the promotion of β-cell regeneration, attenuation of apoptosis and inflammation, and enhancement of islet vascularization (12, 16). Mechanistically , ADSCs mitigate insulin resistance in T2DM by suppressing chronic inflammation via polarization of pro-inflammatory M1 macrophages to anti-inflammatory M2 phenotypes, a process mediated by interleukin-10 (IL-10) and inhibition of NLRP3 inflammasome activation (17, 18) . In T1DM, ADSCs restore immune homeostasis by downregulating pathogenic Th1/Th17 responses and promoting regulatory T-cell (Treg) expansion, thereby attenuating autoimmune β-cell destruction (19, 20) . Molecularly , ADSC-Exos deliver microRNAs (e.g., miR-146a) that inhibit NF-κB and STAT3 signaling, reducing cytokine storms in pancreatic islets (21) . Additionally, ADSCs enhance β-cell survival and function via paracrine secretion of hepatocyte growth factor (HGF) and vascular endothelial growth factor (VEGF) to promote β-cell proliferation and reduce apoptosis (22) . Preclinically , in streptozotocin-induced T1DM rodent models, systemic ADSC administration improves glycemic control by restoring β-cell mass, reducing hyperglycemia (22, 23). In high-fat-diet-induced T2DM mice, ADSCs alleviate insulin resistance via AMPK/SIRT1-mediated enhancement of mitochondrial metabolism in adipose tissue and skeletal muscle (24, 25). Clinically , phase II trials in T2DM patients demonstrate that umbilical cord-derived MSCs (UC-MSCs) reduce HbA1c by 1.5–2.0% and improve insulin sensitivity, correlating with decreased serum TNF-α and increased adiponectin levels (26) . For T1DM, prior case reports have indicated that the infusion of *in vitro*-differentiated insulin-producing cells derived from ADSCs into patients led to sustained stabilization of blood glucose levels and glycosylated hemoglobin (HbA1c) (27), and a prospective dual-arm clinical trial demonstrated that autologous ADSCs combined with bone marrow-derived hematopoietic stem cells (BM-HSCs) enable sustained glycemic regulation (28). Other clinical trials report transient C-peptide preservation and reduced exogenous insulin requirements, though long-term efficacy remains inconsistent (29) . Challenges include donor-dependent variability in ADSC secretome potency, risks of iPSC-derived β-cell teratoma formation due to residual pluripotency , and fibrotic complications linked to TGF-β overactivation during immunomodulation (30) . Emerging strategies to address these limitations involve biomaterial encapsulation for targeted pancreatic delivery and IFN-γ preconditioning to enhance immunosuppressive capacity (31) .

### 2.2 Obesity and metabolic syndrome

Obesity induces a decline in ADSC activity, with studies demonstrating a 40% reduction in ADSC activity in obese populations compared to healthy individuals, directly impairing tissue regenerative capacity and potentially triggering metabolic disturbances such as arteriosclerosis and insulin resistance (32). Under obese conditions, ADSCs exhibit a shift toward a pro-inflammatory secretory profile characterized by increased secretion of IL-1β, IL-6, and IL-8 cytokines, accompanied by a concomitant reduction in immunomodulatory, insulin-sensitizing, and weight-regulatory adipokines (e.g., IL-10, TGF-β, and adiponectin), thereby exacerbating metabolic dysfunction (33–35). And Yu Meng et al.’s study demonstrates that obesity impairs the structural integrity and functional capacity of ADSCs’ mitochondria, potentially mediated in part through miRNA-induced regulation of mitochondrial genes, leading to an escalation in oxidative stress (36). Moreover, studies in diet-induced obesity models have established that PD-L1 upregulation contributes to T cell dysfunction, characterized by a marked impairment in cytolytic activity (37). Beyond investigations in animal models, a clinical study involving 47 reproductive-aged African women demonstrated that serum from overweight/obese individuals (with or without metabolic syndrome) significantly impaired ADSCs proliferation and migration, linked to elevated IL6 levels, and induced lipid accumulation during osteogenic differentiation, highlighting systemic inflammatory dysregulation as a key driver of ADSC functional decline in metabolic disorders (38). Although the reciprocal interactions between obesity and ADSCs remain incompletely elucidated, inspired by existing research, ADSCs may hold broad clinical application prospects in obesity and obesity-related diseases. ADSC-derived exosomes ameliorate obesity-associated metabolic dysregulation by promoting M2 macrophage polarization, enhancing white adipose tissue beiging, and improving insulin sensitivity through STAT3-mediated arginase-1 activation (39). Numerous *in vivo* studies have demonstrated the efficacy of ADSCs in promoting weight loss and ameliorating hyperlipidemia (40–42). Non-alcoholic fatty liver disease (NAFLD), a spectrum ranging from hepatic steatosis to non-alcoholic steatohepatitis (NASH), fibrosis, cirrhosis, and hepatocellular carcinoma, is closely associated with obesity and metabolic syndrome (43, 44). Studies indicate that ADSCs mitigate NAFLD progression by homing to damaged liver tissue, enhancing hepatocyte regeneration, and exerting anti-inflammatory/antioxidant effects through their self-renewal and multipotent differentiation capabilities (45, 46). Although current studies on the mechanisms and preclinical research of ADSCs in obesity and metabolic disorders suggest their potential therapeutic roles, translating these findings into clinical applications remains a protracted process. This transition necessitates rigorous optimization of multiple procedures, including standardized isolation, purification, expansion, and clinical validation of ADSCs, as well as comprehensive safety and efficacy evaluations in human trials.

## 3 Tumor-related application

### 3.1 ADSCs and tumor microenvironment

ADSCs exhibit a multifaceted and bidirectional relationship with the tumor microenvironment (TME), a heterogeneous ecosystem comprising immune cells, stromal fibroblasts, extracellular matrix (ECM), and metabolic mediators that collectively drive tumor progression and therapeutic resistance (47, 48) . A vitro study demonstrates that bidirectional crosstalk between human adipose-derived stem cells (ADSCs) and malignant melanoma cells (MMCs) in an indirect co-culture model significantly enhances tumor-promoting behaviors, including migration, invasion, and angiogenesis, via upregulation of pro-angiogenic factors (VEGF, IL-8, CCL2), matrix metalloproteinases (e.g., MMP-2), and oncogenic mediators (CXCL12, PTGS2, IL-6, HGF) (49, 50). ADSCs are recruited to the TME via tumor-secreted chemotactic signals, such as CCL2 and CXCL12, where they undergo functional reprogramming to adopt pro-tumorigenic roles (49, 51). Within the TME, ADSCs contribute to immunosuppression by secreting anti-inflammatory cytokines (e.g., IL-10, TGF-β) and exosomes that dampen cytotoxic T-cell activity, promote regulatory T-cell (Treg) expansion, polarize macrophages toward an immune-tolerant M2 phenotype, inhibit of dendritic cells differentiation, promoting immune escape of tumor cells (18, 19) . The TME reciprocally shapes ADSC behavior through metabolic crosstalk. Hypoxia and nutrient deprivation in the TME drive ADSCs to release fatty acids and lipid metabolites, which tumor cells exploit to fuel oxidative phosphorylation (OXPHOS) and mitigate metabolic stress (52) . This lipid transfer is further amplified by ADSC-derived exosomal miRNAs (e.g., miR-21, miR-155), which activate oncogenic pathways such as PI3K/Akt and Wnt/β-catenin in adjacent tumor cells (53, 54) . Notably, obesity exacerbates this metabolic symbiosis, as high-fat diets enhance lipid availability in the TME, impairing CD8+ T-cell function and accelerating tumor growth—a mechanism conserved across murine and human cancers (55–57) . Furthermore, a vitro study demonstrates MSCs promote reversible metastatic enhancement in breast carcinoma through TME-driven CCL5/RANTES secretion, which activates CCR5-mediated paracrine signaling to potentiate cancer cell motility, invasion, and distant dissemination (58). In contrast to their tumor-promoting effects, MSCs have also been shown to inhibit the progression of malignancies such as leukemia and hepatocellular carcinoma; therefore, as multipotent stromal cells, MSCs can secrete context-dependent factors within the tumor microenvironment that either suppress or exacerbate neoplastic growth through bidirectional modulation of oncogenic signaling pathways (59). Beyond their established roles in promoting tumorigenesis, progression, and metastasis, ADSCs are also closely associated with chemoresistance to antitumor agents.

### 3.2 ADSCs and chemoresistance

ADSCs drive chemoresistance in malignancies through multifaceted mechanisms within the TME. Primarily, ADSCs engage in bidirectional crosstalk with tumor cells via paracrine signaling, exemplified by the TSG-6/COX-2 axis: Tumor necrosis factor alpha-stimulated gene/protein 6 (TSG-6) upregulates cyclooxygenase-2 (COX-2) expression, fostering prostaglandin E2 (PGE2)-mediated angiogenesis, immunosuppression, and apoptosis evasion, a mechanism validated by the abrogation of chemoresistance upon TSG-6 silencing in ADSCs (60–63). Furthermore , ADSCs secrete interleukin-6 (IL-6), which activates STAT3 in tumor cells to enhance DNA repair and upregulate multidrug resistance (MDR) transporters such as P-glycoprotein (64, 65). Concurrently , metabolic reprogramming induced by ADSCs—such as the release of platinum-induced polyunsaturated fatty acids (PIFAs) to scavenge reactive oxygen species (ROS)—protects tumor cells from cytotoxic damage, a process reversible through COX-1/thromboxane synthase inhibition (66). Additionally , ADSCs remodel the extracellular matrix (ECM) via matrix metalloproteinase (MMP)-mediated stiffening, creating physical barriers that impede drug penetration while promoting hypoxia-driven angiogenesis through HIF-1α/VEGF activation (67). Critically , ADSCs enhance tumor cell stemness by inducing epithelial-mesenchymal transition (EMT) and cancer stem cell (CSC) phenotypes via Wnt/β-catenin and Notch signaling, thereby amplifying self-renewal capacity and therapeutic resistance (68, 69). Notably , targeting these pathways—such as COX-2/PGE2 inhibition, TSG-6 neutralization, or metabolic disruption of PIFA synthesis—holds therapeutic potential to restore chemosensitivity. In summary , ADSCs orchestrate a pro-survival TME through integrated paracrine, metabolic, and structural adaptations, highlighting the urgency of developing stroma-targeted adjuvants to overcome multidrug resistance in oncology.

### 3.3 Tumor related application

Although ADSCs may exhibit pro-tumorigenic effects, including tumor initiation, progression, and metastatic dissemination through crosstalk with the tumor microenvironment, emerging evidence paradoxically highlights their significant clinical potential as anti-neoplastic agents through immunomodulatory mechanisms and targeted therapeutic delivery. Preclinical studies demonstrate their capacity to suppress tumor growth in glioblastoma (70), hepatocellular carcinoma (71, 72), colon cancer (73, 74) and other tumor models (75). Meanwhile, the homing capacity of ADSCs to tumor niches positions them as ideal cellular vectors for targeted drug delivery. Engineered ADSCs overexpressing TNF-α or TRAIL via CRISPR/Cas9 have shown enhanced tumor-specific cytotoxicity in murine models, selectively localizing to metastatic sites while minimizing off-target effects (76, 77). Additionally, ADSC-derived exosomes loaded with chemotherapeutic agents (e.g., doxorubicin) or oncolytic viruses achieve precise intratumoral delivery, overcoming biological barriers and reducing systemic toxicity (78, 79). Recent advances highlight their utility in photodynamic therapy, where photoactivated ADSCs release reactive oxygen species (ROS) to induce localized tumor cell death, synergizing with checkpoint inhibitors to amplify anti-PD-1/PD-L1 efficacy (80). ADSCs, in addition to their direct/indirect antitumor effects, have also demonstrated positive clinical effects in managing toxic side effects associated with chemoradiotherapy, including cisplatin-induced fertility impairment (81), salivary gland dysfunction following radiotherapy for head and neck tumors (resulting in xerostomia) (80, 82), and chemotherapy-induced granulocytopenia through secreting Granulocyte Colony-Stimulating Factor (G-CSF (83, 84). Ongoing research focuses on bioengineering strategies to “lock” ADSCs in anti-tumor states through metabolic priming (e.g., mTOR inhibition) or epigenetic modulation to suppress pro-metastatic gene networks . These innovations underscore ADSCs’ versatility as modular therapeutic platforms, though rigorous characterization of their spatiotemporal dynamics within the TME remains critical to mitigate context-dependent risks.

## 4 Immunomodulatory effects

ADSCs exhibit multifaceted immunomodulatory properties in the treatment of autoimmune diseases and mitigation of post-transplant immune rejection, primarily through paracrine signaling and direct cell-cell interactions. Mechanistically , ADSCs secrete anti-inflammatory cytokines (e.g., IL-10, TGF-β) and induce regulatory T-cell (Treg) expansion while suppressing pro-inflammatory Th17 and effector T-cell activation (19). Additionally, ADSCs upregulate PD-1/PD-L1 interactions to induce T-cell anergy and apoptosis (85). *In vitro* studies support above results and also demonstrate that mesenchymal stem cells (MSCs) exert immunomodulatory effects by suppressing T-cell proliferation, cytotoxicity, and Th1/Th2 cytokine secretion, inhibiting dendritic cell maturation via Notch pathway activation, and attenuating B-cell proliferation and antibody production (86). We examine the latest research progress on the immunoregulatory effects of adipose-derived mesenchymal stem cells in autoimmune disorders and graft-versus-host disease (GVHD).

### 4.1 Autoimmune disorders

Autoimmune disorders, encompassing various chronic organ-specific and systemic diseases caused by immune system malfunction that mistakenly attacks the body’s own cells and tissues, affect approximately 8-10% of the population, resulting in significant health impairments, elevated mortality rates, and substantial medical burdens (87, 88). As summarized in **Table 1**, numerous preclinical and clinical studies have investigated the therapeutic potential of adipose-derived mesenchymal stem cells (ADSCs) in managing various autoimmune disorders. Rheumatoid arthritis (RA), multiple sclerosis (MS), inflammatory bowel disease (IBD), and type 1 diabetes mellitus (T1DM) represent the most prevalent systemic autoimmune conditions globally. These chronic disorders share a common pathogenesis characterized by dysregulated immune responses leading to persistent organ inflammation and progressive tissue damage. Current standard treatments, including non-steroidal anti-inflammatory drugs (NSAIDs), corticosteroids, immunosuppressants, and chemotherapeutic agents such as methotrexate (MTX), often present significant limitations. Given their demonstrated anti-inflammatory properties and immunomodulatory capabilities, ADSCs have emerged as a promising therapeutic alternative for autoimmune diseases, offering the potential to restore immune homeostasis while avoiding the detrimental side effects of conventional pharmacotherapies. This unique biological profile positions ADSC-based therapy as a novel paradigm in the evolving landscape of autoimmune disease management.

**Table 1:** Preclinical and clinical studies in autoimmune disorders.

Disease	Author(year)	NCT number	Clinical Trial phase	Key points	Reference
RA	Álvaro-Gracia, J.M., et al.(2017)	NCT01663116	Phase Ib/IIa	Intravenous infusion of allogeneic expanded ADSCs demonstrated good overall tolerability and potential clinical efficacy in refractory RA	(89)
RA	Vij, R., et al.(2022)	NCT03691909	Phase I/IIa	Single intravenous infusion of autologous ADSCs is safe and effective for improving joint function in active RA patients.	(90)
MS	Fernández, O., et al.(2018)	NCT01056471	Phase I/II	Intravenous infusion of autologous ADSCs is safe and feasible in patients with secondary progressive multiple sclerosis (SPMS)	(91)
IBD	Panés, J., et al.(2016)	NCT01541579	Phase III	Allogeneic expanded ADSCss represent an effective and safe therapeutic approach for complex perianal fistulas in Crohn’s disease patients refractory to conventional or biologic therapies	(92)
IBD	Lightner, A.L., et al.(2020)	Unknown	Phase I	ADSC therapy demonstrates safety, feasibility, and efficacy in the treatment of refractory Crohn’s rectovaginal fistulas	(93)
IBD	de la Portilla, F., et al.(2013)	NCT01372969	Phase I/IIa	*Local injection of allogeneic ADSCs is a simple, safe, and beneficial therapeutic approach for perianal fistulas in Crohn’s disease patients.*	(94)
IBD	Furukawa, S., et al.(2023)	NCT03706456.	Phase III	Expanded allogeneic ADSCs are safe and effective for refractory Crohn’s perianal fistulas.	(95)
T1DM	Dantas, J.R., et al.	NCT03920397	Pilot Study	Allogeneic ADSCs with vitamin D safely preserved β-cells in recent-onset T1DM without immunesuppression	(96)

RA, rheumatoid arthritis; MS, multiple sclerosis; IBD, inflammatory bowel disease; T1DM, type 1 diabetes mellitus.

### 4.2 Graft-versus-host disease

Acute and chronic graft-versus-host disease (GVHD) are common yet challenging clinical complications following allogeneic hematopoietic stem cell transplantation and solid organ transplantation (such as ocular grafts, kidneys, etc.). Given the immunomodulatory and anti-inflammatory properties of adipose-derived mesenchymal stem cells, they hold potential for combating GVHD (97–99). Animal model studies have clarified the effectiveness of ADSCs against bone marrow aplasia in GVHD (100), while another animal study demonstrated that subconjunctival injection of ADSCs significantly improved post-transplant corneal integrity and promoted wound healing, highlighting their clinical potential in this context (101). Beyond animal research, clinical data on ADSCs for GVHD management have also been reported. A single-arm clinical study involving pediatric patients with refractory bronchiolitis obliterans syndrome (BoS) following allogeneic hematopoietic stem cell transplantation (allo-HSCT) showed that ADSCs could be safely administered (102). Additionally, a phase I/II study indicated that combining ADSCs with immunosuppressive therapy is feasible and safe for chronic GVHD post allo-HSCT, with potential positive impacts on disease progression (103). More studies are warranted to understand the potential benefits of ADSCs in GVHD.

## 5 Regenerative medicine and tissue engineering

ADSCs have emerged as a promising therapeutic tool in regenerative medicine and tissue engineering due to their multifaceted mechanisms of action. Mechanistically, ADSCs possess the ability to self-renew and exhibit multipotent differentiation capacity, differentiating into various cell lineages such as fibroblasts, myocytes, chondrocytes, adipocytes, and immune cells (e.g., macrophages or lymphocytes) under specific microenvironmental cues. This versatility endows ADSCs with broad applications in various medical fields.

In plastic surgery and wound repair, ADSCs promote tissue regeneration through paracrine secretion of angiogenic factors (e.g., VEGF, PDGF, bFGF), which stimulate neovascularization (104–106). Additionally, they exhibit immunomodulatory properties by suppressing pro-inflammatory cytokines (TNF-α, IFN-γ) and upregulating anti-inflammatory mediators (IL-10, IL-4) (107–109). These mechanisms facilitate wound healing and tissue repair by enhancing keratinocyte migration and proliferation, modulating extracellular matrix (ECM) remodeling, and suppressing apoptosis (110–112). Animal models of full-thickness skin defects and ischemic flaps have demonstrated accelerated wound closure, increased capillary density, and reduced fibrosis in ADSC-treated groups, attributed to enhanced angiogenesis and attenuated oxidative stress (113–115). ADSCs have also shown promise in repairing radiation-induced/UV-induced tissue damage (116–119), chronic diabetic ulcers (120–123), and in aesthetic procedures such as facial volumization and breast reconstruction, where their adipogenic potential of improving graft retention and reducing scar formation (124–128).

In osteochondral repair, ADSCs exhibit robust osteogenic potential primarily through paracrine signaling, immunomodulation, and direct differentiation. Paracrine activity enables ADSCs to secrete osteoinductive factors, including BMPs, VEGF, and IGF-1, which synergistically enhance angiogenesis and osteoblast differentiation (129). ADSC-derived exosomes deliver osteogenic miRNAs that activate Wnt/β-catenin and BMP/Smad pathways, upregulating master regulators Runx2 and Osterix (130–133). Furthermore, ADSCs can modulate the immune system to inhibit osteoclastogenesis in conditions like osteoporosis and foster a pro-regenerative microenvironment in osteoarthritic joints (134). Under osteoinductive conditions, ADSCs directly differentiate into osteoblasts, marked by upregulated ALP, collagen type I, and OCN expression, culminating in mineralized matrix deposition (3, 135). These mechanisms have been corroborated across diverse preclinical models, with *in vitro* and *in vivo* studies demonstrating enhanced bone formation and reduced osteoclast activity.

ADSCs also exhibit therapeutic potential in myocardial repair and regeneration. Through paracrine secretion of bioactive molecules and exosomes enriched with cardioprotective miRNAs, ADSCs attenuate oxidative stress, activate PI3K/Akt and HIF-1α pathways, enhance cardiomyocyte survival, and stimulate angiogenesis (136–139). Furthermore, ADSCs can modulate the phenotype of monocytes/macrophages towards a more anti-inflammatory state, potentially beneficially influencing the duration and intensity of the inflammatory response post-myocardial infarction (140). Preclinical studies in rodent models have revealed improvements in left ventricular ejection fraction, reduction in infarct size, and increased amount of highly vascularized granulation tissue in the border zone (141).

In neural repair and regeneration, ADSCs possess inherent multipotency, characterized by their trilineage differentiation potential and robust secretion of neurotrophic mediators. These mediators orchestrate neuroprotection, axonal sprouting, and synaptogenesis in models of neural trauma (142–145). The immunomodulatory axis of ADSCs attenuates neuroinflammation and microglial activation by downregulating pro-inflammatory cytokines and upregulating anti-inflammatory interleukin-10 (146, 147). *In vitro*, ADSCs exhibit neurogenic differentiation potential under inductive conditions, evidenced by upregulation of neuronal markers and electrophysiological properties indicative of functional neuronal networks (148–150). Preclinical *in vivo* studies have demonstrated enhanced functional recovery through various signaling pathways and modulation of glial scar formation (151–154).

Moreover, ADSCs and their bioactive derivatives exhibit multifaceted anti-aging and regenerative potential. At the molecular level, ADSCs modulate senescence-associated pathways via paracrine signaling, releasing exosomes enriched with miRNAs, growth factors, and anti-inflammatory cytokines (105, 108, 155, 156). These components collectively suppress oxidative stress, inhibit the senescence-associated secretory phenotype, and enhance mitochondrial biogenesis. Studies have explored the role of ADSCs in aging-related conditions such as skin aging, alopecia, and cognitive dysfunction, with promising results suggesting potential for mitigating age-related inflammation, improving skin elasticity, and wound healing (157, 158).

## 6 In genetic engineering: applications and prospects 

ADSCs have garnered significant interest in genetic engineering due to their inherent plasticity, ease of isolation, and robust expansion potential. Gene-editing technologies, such as CRISPR/Cas9, TALENs, and viral/non-viral vector systems, enable precise manipulation of ADSCs to enhance their therapeutic efficacy or confer novel functionalities. Genetic modification of ADSCs enhances their therapeutic paracrine signaling by co-upregulating regenerative/immunoregulatory factors (e.g., VEGF, HGF, IL-10) while suppressing pro-inflammatory cytokine expression, thereby amplifying paracrine-mediated tissue regeneration through balanced immunomodulation and matrix remodeling (159). For instance, CRISPR-mediated upregulation of VEGF in ADSCs has demonstrated enhanced angiogenesis in preclinical models of ischemic cardiomyopathy and diabetic wounds. Similarly, ADSCs engineered to express neurotrophic factors (e.g., brain-derived neurotrophic factor, BDNF) show promise in neural regeneration by promoting axonal growth and synaptic plasticity in spinal cord injury models (160). Another emerging application involves modifying ADSCs to improve their homing efficiency and survival in hostile microenvironments. Knockdown of pro-apoptotic genes (e.g., BAX) or overexpression of chemokine receptors (e.g., CXCR4) via lentiviral transduction enhances their engraftment at injury sites (161, 162). Furthermore, ADSCs can be reprogrammed to act as targeted delivery vehicles for therapeutic genes or RNA-based therapies (163, 164). For example, ADSCs transfected with oncolytic viruses or tumor-suppressor genes (e.g., p53, PTEN) exhibit synergistic anti-tumor effects by selectively localizing to tumor stroma and inducing apoptosis in malignant cells while sparing healthy tissues (165). The integration of synthetic biology platforms with ADSCs opens avenues for dynamic, condition-responsive therapies. Engineered gene circuits, such as hypoxia-inducible promoters or inflammation-sensitive switches, allow ADSCs to autonomously release therapeutic payloads (e.g., anti-inflammatory cytokines, matrix metalloproteinase inhibitors) in response to disease-specific cues. This approach minimizes off-target effects and maximizes spatiotemporal precision, as demonstrated in rheumatoid arthritis models where IL-1β-responsive ADSCs suppressed joint inflammation only in active disease states (166). Prospectively, ADSCs hold transformative potential in addressing genetic disorders through ex vivo gene correction. Autologous ADSCs edited to rectify monogenic mutations (e.g., in COL1A1 for osteogenesis imperfecta or CFTR for cystic fibrosis) could be reimplanted to restore functional tissue homeostasis (167). Additionally, ADSCs engineered to express chimeric antigen receptors (CARs) or T-cell engagers are being explored in adoptive cell therapies for cancers, leveraging their tumor-tropic properties to enhance localized immune activation. Despite these advancements, challenges persist in ensuring the safety and scalability of genetically modified ADSCs. Risks of off-target edits, immune rejection of engineered cells, and long-term genomic instability necessitate rigorous preclinical validation. Moreover, standardized protocols for GMP-compliant manufacturing, quality control of edited clones, and regulatory frameworks for clinical translation remain underdeveloped. Collaborative efforts among geneticists, bioengineers, and clinicians are critical to overcoming these barriers and unlocking the full potential of ADSCs in next-generation gene therapies. In summary, ADSCs serve as a versatile platform for genetic engineering, bridging regenerative medicine and precision therapeutics. Their applications span targeted gene delivery, dynamic microenvironment modulation, and autologous cell-based gene correction, with translational prospects in oncology, degenerative diseases, and genetic disorders. Continued innovation in gene-editing tools and biofabrication technologies will likely propel ADSCs to the forefront of personalized and programmable medicine. 

Despite their therapeutic promise in metabolic regulation, immunomodulation, and tissue regeneration (as exemplified by key studies summarized in **Table 2**), the clinical application of ADSCs faces several critical challenges. First, heterogeneity in cell populations derived from the stromal vascular fraction (SVF) and variability in isolation/expansion protocols compromise batch-to-batch consistency, potentially affecting therapeutic reproducibility. Standardized Good Manufacturing Practice (GMP)-compliant protocols, coupled with advanced single-cell sequencing to identify subpopulation-specific biomarkers, may enhance quality control. Second , long-term safety concerns persist, particularly regarding the potential pro-tumorigenic effects of ADSCs in pre-existing malignancies or their unintended differentiation post-transplantation. Rigorous preclinical studies using lineage-tracing models and tumor-prone animal cohorts, alongside real-time molecular monitoring in clinical trials, are essential to elucidate these risks. Third , the pleiotropic mechanisms underpinning ADSCs’ efficacy—such as paracrine signaling, exosome-mediated communication, and dynamic crosstalk with immune cells—remain incompletely mapped. Integrated multi-omics approaches (e.g., proteomic profiling of secretomes and CRISPR-based functional screens) could clarify dominant pathways like PI3K/AKT and Wnt/β-catenin, enabling targeted therapeutic optimization. Fourth , clinical translation is hindered by a paucity of robust Phase III randomized controlled trials (RCTs) and suboptimal delivery routes (e.g., systemic infusion vs. localized injection). Adaptive trial designs, route-specific pharmacokinetic studies, and international consortium-led registries may accelerate evidence generation. By addressing these challenges through interdisciplinary innovation and rigorous regulatory oversight, ADSCs could transition from experimental therapies to standardized clinical tools.

**Table 2:** Studies covering five key aspects related to this review.

Scope	Disease	Author(year)	NCT number	Research stage	Key points	Reference
Metabolic Regulation	DM	Zang, L., et al. (2022)	NCT02302599	Phase II	MSCs transplantation could be a potential therapeutic approach for Chinese adults with T2DM	(26)
	T1DM	Dantas, J.R., et al.(2021)	NCT03920397	Pilot Study	Allogeneic ADSCs with vitamin D safely preserved β-cells in recent-onset T1DM without immune suppression	(96)
Tumor-related Application	Radiation-Induced Hyposalivation in HNC	Fenger Carlander, A.L., et al.(2025)	Unknown	Phase II	Intraglandular ADSC therapy for the submandibular gland can significantly alleviate subjective xerostomia symptoms. However, based on salivary flow rate measurements, ACS is not superior to placebo in restoring salivary gland function.	(168)
	Cisplatin-induced azoospermia and testicular damage	Ismail, H.Y., et al. (2023)	/	Animal model	ADSC therapy ameliorated cisplatin-induced testicular damage, improving biochemical and pathological parameters	(81)
Immunomodulatory Effects	RA	Vij, R., et al.(2022)	NCT03691909	Phase I/IIa	Single intravenous infusion of autologous ADSCs is safe and effective for improving joint function in active RA patients.	(90)
	MS	Fernández, O., et al.(2018)	NCT01056471	Phase I/II	Intravenous infusion of autologous ADSCs is safe and feasible in patients with secondary progressive multiple sclerosis (SPMS)	(91)
	IBD	Furukawa, S., et al.(2023)	NCT03706456.	Phase III	Expanded allogeneic ADSCs are safe and effective for refractory Crohn’s perianal fistulas.	(95)
Regenerative medicine	Knee Osteoarthritis	Dantas, J.R., et al. (2023)	NCT03990805	Phase III, RCT	Intra-articular injection of autologous culture-expanded ADSCs provided significant pain relief and functional improvements in patients with osteoarthritis	(169)
	ischemic cardiomyopathy	Kawamura, T., et al.(2024)	NCT04695522	Phase I	ADSC spray therapy combined with CABG demonstrated safety and efficacy at enhancing cardiac function	(170)
	Acute Ischemic Stroke	de Celis-Ruiz, E., et al.(2022)	Unknown	Phase II	During the 24-month follow-up, intravenous administration of ADSCs within 2 weeks after ischemic stroke was safe and showed a trend toward improving post-stroke sequelae.	(171)
Genetic Engineering	Glioblastoma	Oraee-Yazdani, S., et al. (2023)	Unknown	Phase I	Suicide gene therapy using allogeneic ADSCs carrying the HSV-TK gene is safe in patients with recurrent glioblastoma.	(163)
	NAFLD	Lopez-Yus, M., et al.(2023)	/	Vitro Study	Precise CRISPR/Cas9-mediated genomic editing of ADSCs in the adipocyte-hepatocyte metabolic axis significantly suppresses lipid accumulation in hepatocytes.	(172)

DM, diabetes mellitus; T1DM, Type I diabetes mellitus; HNC, head and neck cancer; RA, rheumatoid arthritis; MS, multiple sclerosis; IBD, inflammatory bowel disease; RCT, Randomized, Placebo-Controlled Trial; CABG, Coronary artery bypass grafting; NAFLD, nonalcoholic fatty liver disease.
